# The Impact of Glucagon-Like Peptide-1 Receptor Agonist–Induced Nutritional Deficiencies on Skin Health: A Narrative Review With a Proposed Framework for the Aesthetic Practitioner

**DOI:** 10.1093/asjof/ojag169

**Published:** 2026-09-11

**Authors:** Justin Karlin, Zhaoping Li, Kami Parsa

## Abstract

Semaglutide and tirzepatide have reshaped obesity care, and aesthetic practitioners increasingly encounter patients with rapid-weight-loss-associated facial volume loss and skin changes. Nutritional deficiency may be an underrecognized contributor to impaired dermal repair during glucagon-like peptide-1 receptor agonist (GLP-1RA) therapy. The authors reviewed and synthesized evidence across metabolic pharmacology, facial anatomy and aesthetics, dermatologic histology, and clinical nutrition. PubMed, Embase, and the Cochrane Library were searched from January 2019 to March 2026, supplemented by foundational studies in collagen biology and post-weight-loss skin histology. GLP-1RA-mediated caloric restriction can reduce intake of micronutrient cofactors required for collagen biosynthesis and wound repair. Vitamin D deficiency reaches 13.6% at 12 months, and ∼22.4% of patients have at least one documented deficiency within 1 year. Deficiencies in vitamin D, iron, vitamin B12, folate, zinc, copper, and protein may plausibly compound rapid fat-compartment depletion and collagen remodeling. Direct GLP-1RA effects on dermal cells remain preclinical and should be interpreted cautiously. In summary, GLP-1RA-associated facial aging changes are multifactorial. The authors propose a risk-stratified framework for aesthetic practitioners emphasizing dietary optimization, selective supplementation, targeted laboratory assessment in at-risk patients, coordination with prescribing clinicians, and procedural timing when nutritional status is optimized. Prospective studies are needed to determine whether correcting deficiencies improves aesthetic or surgical outcomes.

**Level of Evidence:** 5 (Therapeutic) 
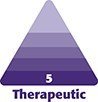

Semaglutide and the dual glucagon-like peptide-1 receptor agonist (GLP-1RA) and glucose-dependent insulinotropic polypeptide (GIP) agonist tirzepatide produce weight reductions of 15% to 21% in clinical trials, and the 2025 World Health Organiztion guideline now endorses their use for obesity management.^1-4^

Although these drugs may reduce the risk of obesity-related disease, the rapid weight loss they cause may detrimentally impact facial aesthetics. Fat compartments deflate, dermal collagen remodels, and lean body mass declines.^5-8^ The colloquial term “Ozempic face” captures the result: temporal wasting, malar flattening, deepened nasolabial folds, and periorbital hollowing that accompany GLP-1RA-induced weight reduction.^9-11^ The underlying pathophysiology is not fat loss alone. Subcutaneous fat depletion, dermal white adipose tissue (dWAT) atrophy, collagen disorganization, and nutritionally driven impairment of extracellular-matrix repair each contribute.

GLP-1RA therapy may create a state in which tissue-remodeling demand increases while nutritional substrate availability declines, a “nutritional–dermal mismatch.” The appetite-suppressive mechanism can reduce caloric intake by one-quarter to one-third relative to baseline, with some patients consuming below 800 kcal/day.^12-15^ An extreme calorie-restricted diet in these patients may rapidly deplete the cofactors required for collagen biosynthesis, cross-linking, and dermal repair—including vitamin D, vitamin C, zinc, iron, copper, and the B vitamins (Figure 1).^16-18^

**Figure 1.**
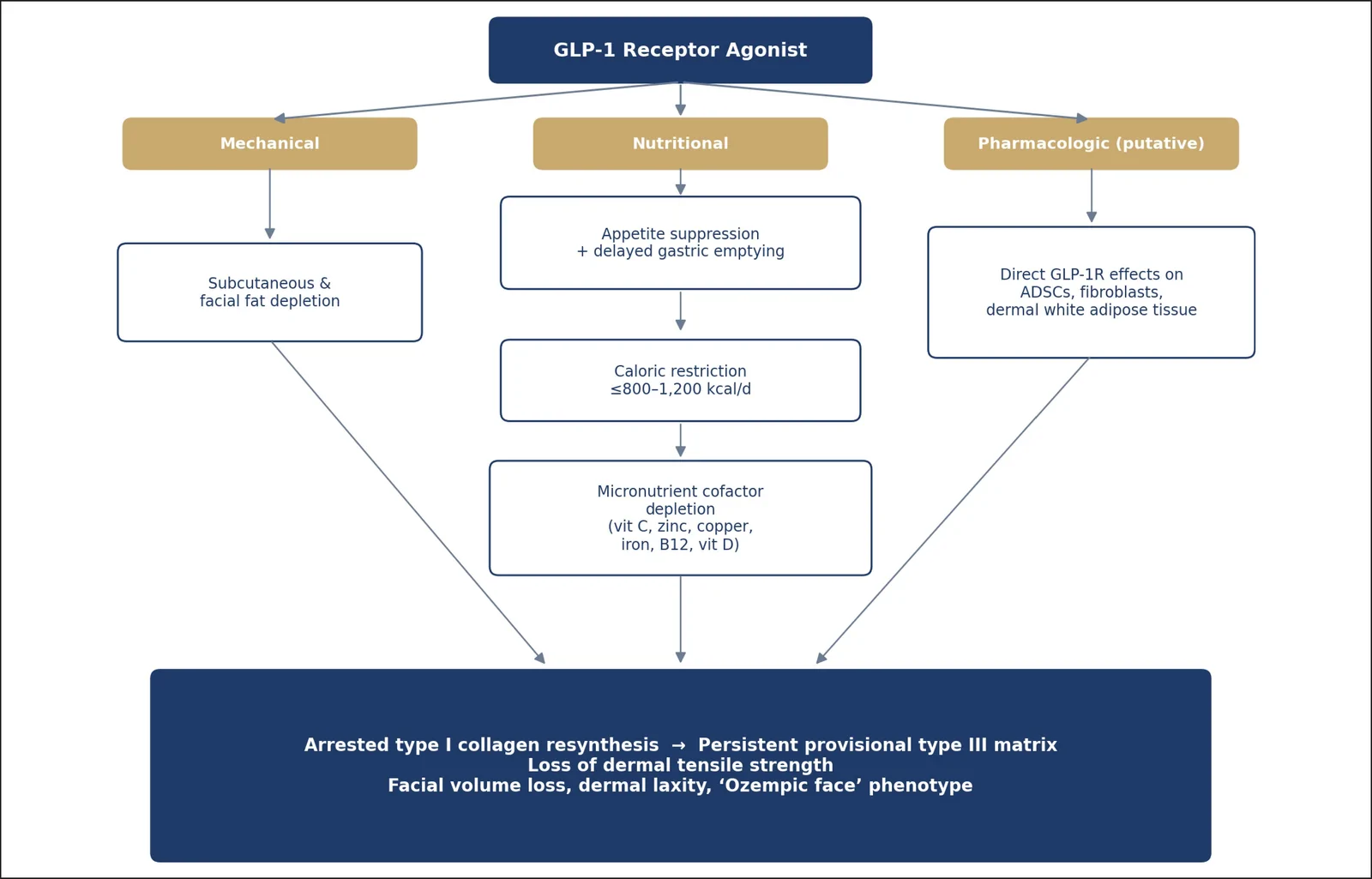
Mechanistic schematic of the nutritional–dermal mismatch. GLP-1 receptor agonist therapy may contribute to facial aging through 3 convergent pathways: mechanical fat depletion, nutritional cofactor depletion secondary to appetite suppression and caloric restriction, and putative direct receptor-mediated effects on adipose-derived stem cells, fibroblasts, and dermal white adipose tissue. These pathways converge on impaired type I collagen resynthesis, persistent provisional type III matrix, loss of dermal tensile strength, facial volume loss, and dermal laxity. ADSC, adipose-derived stem cell; GLP-1R, glucagon-like peptide-1 receptor; GLP-1RA, glucagon-like peptide-1 receptor agonist.

Aesthetic practitioners are often the first to evaluate patients with visible facial sequelae of rapid weight loss and are well positioned to identify nutritional contributors and coordinate care. This review therefore has 3 aims: (1) to summarize the micronutrient deficiencies most commonly associated with GLP-1RA therapy; (2) to examine the dermatologic and histological consequences of GLP-1RA-induced rapid weight loss, including the possible contribution of direct receptor-mediated effects on dermal cells; and (3) to propose a risk-stratified framework integrating nutritional awareness into aesthetic practice.

## METHODS

This narrative review was conducted in accordance with SANRA criteria.^19^ PubMed, Embase, and the Cochrane Library were searched from January 2019 to March 2026 (final update March 28, 2026). The search combined terms across 3 conceptual domains: (1) drug exposure—“GLP-1 receptor agonist,” “semaglutide,” “liraglutide,” “tirzepatide,” “dulaglutide,” “exenatide,” “incretin mimetic,” and “GLP-1RA”; (2) nutritional and metabolic effects—“micronutrient deficiency,” “vitamin deficiency,” “zinc,” “iron,” “vitamin C,” “vitamin D,” “ascorbic acid,” “sarcopenia,” “lean mass loss,” “body composition,” “malnutrition,” “rapid weight loss,” “caloric restriction,” and “protein deficiency”; and (3) cutaneous and facial outcomes—“skin aging,” “collagen,” “collagen biosynthesis,” “dermal atrophy,” “facial volume loss,” “facial aging,” “Ozempic face,” “dermal white adipose tissue,” “dWAT,” “elastin,” “extracellular matrix,” “periorbital hollowing,” “fat atrophy,” and “facial aesthetics.” Boolean operators combined terms within (OR) and between (AND) domains. Reference lists of included articles and relevant reviews were hand searched. Preclinical, clinical, and epidemiologic study designs were eligible. Non-English articles were excluded during screening because dual-reviewer translation resources were not available; this is acknowledged as a limitation. Two authors (J.K. and K.P.) independently screened titles and abstracts; disagreements were resolved by discussion with the co-author (Z.L.). Searches yielded 1284 unique records; 312 underwent full-text review; 45 primary sources are cited in the synthesis. Data were synthesized descriptively per narrative review format.

## RESULTS

### The “Ozempic Face” Phenotype

GLP-1 receptor agonists suppress appetite through hypothalamic signaling and delayed gastric emptying.^20^ Semaglutide achieves sustained receptor activation through albumin binding and dipeptidyl peptidase-4 (DPP-4) resistance; tirzepatide, a dual GLP-1/GIP agonist, produces greater weight reductions.^2,21^

Patients taking GLP-1RA predominantly lose fat mass, but clinically significant lean-mass loss occurs in parallel. In the STEP-1 trial, ∼40% of total weight loss was lean mass.^22^ The SEMALEAN study (*n* = 115) confirmed 13% weight loss at 12 months with semaglutide 2.4 mg, with lean mass declining by 3 kg in the first 7 months before stabilizing; 49% exhibited sarcopenia at baseline.^23^ A systematic review confirmed notable lean-mass losses in larger trials, and a retrospective cohort of 220 older adults identified semaglutide dosage, baseline muscle mass, and gait speed as independent predictors of muscle loss.^22,24^ Skeletal muscle provides the structural scaffold upon which facial and cervical soft tissue is draped; sarcopenia exacerbates the visual impact of fat depletion and skin laxity.^6,25^

Facial fat-compartment depletion is the most visible consequence of rapid weight loss. The face contains distinct superficial and deep fat compartments, each with independent vascular supply and unique susceptibility to volume loss.^26^ Jafar et al documented midcheek hollowing in 88% of massive-weight-loss patients, with patients appearing up to 5 years older.^27^ Sharma et al provided the first quantitative 3-dimensional (3D) radiographic analysis, demonstrating a median 9.0% decrease in total midfacial volume, with superficial fat pads losing 11.0%, a change that correlated linearly with weight loss (∼7% of midfacial volume per 10 kg lost), whereas deep sub-superficial musculoaponeurotic system (sub-SMAS) fat compartments were relatively preserved.^28^ The clinical pattern is predictable and compartment-specific: superficial temporal fat pad deflation produces temporal hollowing, malar fat pad atrophy deepens the nasojugal groove and flattens the midface, and nasolabial fat loss accentuates the nasolabial fold; periorbital changes may include periocular hollowing.^29^ The velocity of weight loss may independently compound these changes: rapid deflation can outpace the skin's capacity for elastin-mediated recoil and collagen remodeling, producing tissue ptosis and dermal redundancy disproportionate to the absolute volume lost.^28^ Demographic modifiers, such as age, sex, baseline BMI, and Fitzpatrick phototype, likely moderate susceptibility, although comparative data remain sparse.

### The Nutritional–Dermal Mismatch

Urbina et al synthesized data from 6 studies (480,825 adults) and identified vitamin D deficiency as the most common abnormality, reaching 7.5% at 6 months and 13.6% at 12 months.^25^ Melis et al demonstrated impaired intestinal iron absorption with semaglutide.^30^ Case reports have linked GLP-1RA use to Wernicke encephalopathy and dry beriberi.^31,32^

Butsch et al found ∼22.4% of patients had at least one deficiency diagnosed within 1 year.^32^ This figure derives from claim-based codes rather than universal screening; subclinical insufficiency is likely more prevalent. Johnson et al and Christensen et al reported caloric intake frequently approaching 800 kcal/day, and none of the pivotal trials reported micronutrient intake data.^14,15^

Bariatric surgery (particularly procedures such as Roux-en-Y gastric bypass) causes nutritional deficiencies through predictable anatomic alteration of gastrointestinal absorption, and structured monitoring protocols are thus well established.^33^ GLP-1RA therapy, in contrast, operates through a restrictive mechanism (appetite suppression and delayed gastric emptying) rather than through changing nutrient absorption. Unlike bariatric patients, those on GLP-1RAs are seldom monitored systematically for intake-driven deficiencies that may result from sustained caloric restriction. A 2025 multisociety advisory established nutritional priorities for GLP-1RA therapy, emphasizing protein intake, micronutrient monitoring, and dietary quality.^34^ Almandoz et al recommended nutritional assessment during antiobesity medication therapy, emphasizing 3 principles directly relevant to the present review: (1) identify preexisting nutritional risk factors before initiating antiobesity medications, not after sequelae manifest; (2) dietary quality becomes paramount when total caloric intake is pharmacologically reduced, because every calorie must carry greater micronutrient density; and (3) ongoing monitoring of protein adequacy, fiber intake, micronutrient status, and fluid balance should be standard during active therapy.^35^ Key micronutrient deficiencies and their dermatologic relevance are summarized in Table 1.

**Table 1.** Key Micronutrient Deficiencies, Dermatologic Relevance, and Quantitative Gap at 800 kcal/Day

Micronutrient	Role in dermal health	RDA (adult)	Typical intake at ∼800 kcal/day (% RDA)	Prevalence in GLP-1RA cohorts	Key reference
Vitamin D	Keratinocyte differentiation, barrier function, immune modulation	600-800 IU	∼20%-40%	7.5% at 6 months; 13.6% at 12 months	Urbina et al (2026)^25^
Iron	Prolyl hydroxylase cofactor; O_2_ transport	8-18 mg	∼40%-60%	Impaired absorption documented	Melis et al (2025)^30^
Vitamin B1 (thiamine)	Cellular energy metabolism	1.1-1.2 mg	∼50%-70%	Wernicke encephalopathy reported	Sharma et al (2024)^31^
Vitamin B12	DNA synthesis; neurologic function	2.4 µg	Variable	Risk with prolonged restriction	Urbina et al (2026)^25^
Vitamin C	Prolyl/lysyl hydroxylase cofactor	75-90 mg	∼30%-50%	Subclinical depletion likely <800 kcal/d	Johnson et al (2025)^14^
Zinc	Collagenase activation; cross-linking	8-11 mg	∼40%-55%	Subclinical depletion likely <800 kcal/d	Johnson et al (2025)
Copper	Lysyl oxidase cofactor; elastin cross-linking	0.9 mg	∼30%-50%	Under-reported; risk with prolonged restriction	Harris et al (1980), Almandoz et al (2024)^35^
Manganese	Lysyl oxidase cofactor	1.8-2.3 mg	∼40%-60%	Under-reported	Harris et al (1980)^36^

Key micronutrient deficiencies, dermatologic relevance, and estimated intake gap at severely restricted caloric intake. Typical intake estimates derive from published dietary analyses in GLP-1RA cohorts and general nutrition databases; values are approximate and highlight the magnitude of the intake–demand gap rather than individual clinical assessments. GLP-1RA, glucagon-like peptide-1 receptor agonist; RDA, Recommended Dietary Allowance.

The micronutrients most commonly affected during GLP-1RA therapy are those required for collagen biosynthesis. Vitamin C is essential for prolyl and lysyl hydroxylase.^16,37^ Zinc is required for collagenase activation and cross-linking; deficiency can reduce collagen production by up to 80%.^17,38^ Vitamin D regulates keratinocyte differentiation and dermal inflammation.^38^ Iron serves as a prolyl hydroxylase cofactor.^39^ Vitamin A stimulates fibroblast collagen synthesis while suppressing matrix metalloproteinase (MMP) mediated degradation.^40^ Copper and manganese are cofactors for lysyl oxidase.^36^

At intake of 800 kcal/day, typical achieved micronutrient provision falls below 50% of the Recommended Dietary Allowance for vitamin C, zinc, and copper in most published intake analyses, whereas baseline fibroblast cofactor demand for routine dermal collagen turnover is unchanged and may be elevated during active tissue remodeling.^14,15,35^ Nearly every step from procollagen synthesis to mature fiber cross-linking therefore depends on a micronutrient for which GLP-1RA patients may be at increased risk of insufficiency (Table 2).

**Table 2.** Quantitative Cofactor Demand vs Intake at 800 kcal/Day

Cofactor/process	RDA or steady-state requirement	Typical intake at ∼800 kcal/day	Estimated % of requirement met
Baseline dermal collagen turnover (adult)	∼1%-2% of total dermal collagen turned over per day	Not applicable	Demand rises further during active tissue remodeling
Vitamin C (prolyl/lysyl hydroxylation)	≥75 mg/day	25-45 mg/day	∼30%-60%
Zinc (lysyl oxidase cross-linking)	8-11 mg/day	3-6 mg/day	∼40%-55%
Copper (lysyl oxidase cofactor)	0.9 mg/day	0.3-0.5 mg/day	∼35%-55%
Protein (procollagen synthesis)	1.2-1.6 g/kg/day (during active weight loss)	Often <0.8 g/kg/day	<65% (approximate; substantial individual variation)

Illustrative substrate-demand estimates for dermal collagen turnover during GLP-1RA-induced caloric restriction at ∼800 kcal/day. Baseline dermal collagen turnover is ∼1% to 2% of total dermal collagen per day in adults; cofactor demand for accelerated remodeling likely exceeds these steady-state estimates. Intake estimates are derived from published dietary analyses of GLP-1RA cohorts and USDA FoodData Central median intakes at matched energy levels; individual variation is substantial.^14,15,35^ GLP-1RA, glucagon-like peptide-1 receptor agonist; RDA, Recommended Dietary Allowance.

The consequences of weight loss on skin histology have been well documented. Rocha (2021) found that post-weight-loss skin showed a significant increase in thin type III collagen fibers and a corresponding decrease in thick, organized type I fibers, whereas total collagen content remained unchanged.^41^ Type III and type I collagen are separate proteins encoded by different genes (COL3A1 and COL1A1). Type III does not mature into type I. During normal remodeling, the body deposits type III collagen as a rapid provisional scaffold, then degrades it through matrix metalloproteinases and replaces it with structurally superior type I collagen. Rocha et al observed that, in individuals after weight loss, there is an “arrested” version of this process: matrix metalloproteinase-driven degradation of load-bearing type I collagen proceeds, but resynthesis of mature collagen does not keep pace, leaving the dermis populated with a provisional type III matrix that lacks the tensile strength of the collagen type I it replaced.

The synthesis of new type I collagen to replace the provisional type III matrix requires active fibroblast production of procollagen, which must be hydroxylated by prolyl hydroxylase (requiring vitamin C and iron), stabilized into its triple-helix conformation, and cross-linked by lysyl oxidase (requiring copper and zinc) to achieve mature load-bearing capacity. If these cofactors are depleted during the active remodeling window, the transition from provisional to mature collagen may stall, prolonging structural vulnerability.

Dietary protein sources (eg, lean meats, fish, eggs, dairy, legumes, and nuts) provide not only the glycine, proline, and hydroxyproline precursors for endogenous collagen synthesis but also the broader array of micronutrient cofactors (vitamin C, zinc, iron, and copper) that support every step of the collagen maturation pathway. Dietary sources therefore offer a synergistic advantage that isolated supplements cannot replicate, and dietary optimization should be considered the foundational intervention for supporting dermal repair during GLP-1RA therapy.

When dietary protein intake is limited by GLP-1RA-mediated appetite suppression, oral nutrition supplements (high-protein medical foods or fortified shakes, typically 15 to 30 g protein per serving) and oral hydrolyzed collagen peptides may warrant consideration as adjunctive substrate sources. These 2 strategies may act synergistically: the oral nutrition supplement supplies a complete amino acid profile together with a broader micronutrient cofactor matrix, whereas hydrolyzed collagen peptides supply targeted glycine, proline, and hydroxyproline precursors and bioactive di- and tripeptide signaling molecules. Upon ingestion, the collagen peptides are absorbed as di- and tripeptides—primarily prolyl-hydroxyproline and hydroxyprolyl-glycine—that function both as substrates for endogenous collagen synthesis and as signaling molecules that stimulate fibroblast proliferation and collagen gene expression.^42^ The 2026 umbrella review by Ravindran et al synthesized 16 meta-analyses encompassing 113 randomized controlled trials (*n* = 7983) and found that oral collagen supplementation was associated with improvements in skin elasticity and hydration, with meta-regression demonstrating dose- and duration-dependent effects.^43^ Supplementation did not significantly improve skin roughness, suggesting benefits are mediated primarily through dermal matrix hydration and elastic remodeling rather than surface texture modification. Collagen supplementation without adequate micronutrient cofactors may yield suboptimal results; conversely, cofactor repletion without sufficient protein substrate is similarly incomplete.

Studies advocating for collagen supplementation must be interpreted with caution: only 1 of 16 included meta-analyses was rated high quality by AMSTAR 2 criteria, many trials were industry-funded, and no studies have evaluated collagen supplementation specifically in the GLP-1RA population.^43^ Oral collagen peptides (5-15 g daily, co-administered with vitamin C) may therefore be considered 1 option within a comprehensive dietary strategy for patients whose protein intake remains inadequate despite counseling but should not be positioned as routine.

### Direct Receptor-Mediated Effects on Dermal Cells

Beyond nutritional mechanisms, preclinical evidence suggests that GLP-1RAs may directly affect dermal homeostasis through receptor-mediated pathways. GLP-1 receptor expression has been reported in adipose-derived stem cells (ADSCs), fibroblasts, and sebocytes.^44,45^ Paschou et al reviewed evidence that in vitro receptor stimulation reduces ADSC protective cytokine production, promotes reactive oxygen species generation, decreases ADSC glucose uptake (leading to apoptosis), and indirectly reduces estrogen production from dWAT, diminishing fibroblast collagen synthesis.^44^ Ridha et al similarly concluded that accelerated facial aging in this population is multifactorial, involving loss of dWAT and subcutaneous white adipose tissue, altered ADSC proliferation and differentiation, and diminished facial muscle mass.^8^

Two caveats regarding the direct effects of GLP-1RA on dermal homeostasis are worth mentioning. First, all dermal data are in vitro and typically employ supraphysiologic agonist concentrations; steady-state plasma concentrations of semaglutide at therapeutic doses (∼65-120 nmol/L) are well below those used in most in vitro studies, and dermal tissue partitioning of these large peptide agonists is poorly characterized. Second, receptor expression in human facial skin has not been systematically mapped across anatomic subunits or Fitzpatrick types. These preclinical findings therefore raise, but do not yet establish, the possibility that GLP-1RA-associated facial changes represent a distinct clinical entity, driven by the convergence of rapid fat-compartment deflation, direct receptor-mediated effects on dermal progenitor cells, and nutritional substrate depletion, rather than simply an accelerated form of weight-loss aging.

### GLP-1RA vs Bariatric Surgery vs Voluntary Very-Low-Calorie Diet

The facial and nutritional phenotypes of rapid weight loss share substantial overlap across etiologies, and the drug specificity of “Ozempic face” cannot be assumed. Table 3 summarizes current evidence contrasting GLP-1RA therapy, bariatric surgery, and voluntary very-low-calorie diet across mechanism, rate and composition of weight loss, dominant nutritional pattern, and characteristic facial findings. Until prospective studies compare facial outcomes in GLP-1RA patients against weight-matched controls who lost equivalent mass through other routes, the relative contributions of pharmacologic vs purely weight-loss-driven mechanisms remain indeterminate.

**Table 3.** Comparator Framing: GLP-1RA vs Bariatric Surgery vs Voluntary VLCD

Feature	GLP-1RA	Bariatric surgery (RYGB/SG)	Voluntary VLCD (<800 kcal/day)
Primary mechanism	Restrictive (appetite suppression, delayed gastric emptying)	Restrictive + malabsorptive (anatomic)	Restrictive (behavioral)
Typical total weight loss	15%-21% at 12-18 months	25%-35% at 12-24 months	10%-20% at 3-6 months (often rebounds)
Rate of weight loss	Moderate to rapid; exposure-dependent	Rapid (first 6-12 months)	Rapid
Lean-mass loss proportion	∼25%-40% of total loss	∼20%-30%	Often >30% without resistance training
Dominant nutritional risks	Intake-driven: vitamin C, zinc, B12, vitamin D, iron absorption	Malabsorptive: B12, iron, Ca, fat-soluble vitamins	Intake-driven: protein, vitamin C, iron
Monitoring standard of care	Not systematized	Well established, guideline-driven	Ad hoc
Dermal/facial phenotype	Temporal/malar deflation; periorbital hollowing; dermal laxity; possible direct GLP-1R effect	Skin laxity dominant; significant redundancy; often surgical candidates	Similar laxity pattern without drug-specific dermal effect
Key references	^5, 8, 22, 23, 25, 28, 32, 44^	^33^	^41^

Comparator framing across rapid-weight-loss modalities. Apparent drug specificity of facial changes in GLP-1RA users should be interpreted against overlapping phenotypes in bariatric and VLCD populations. GLP-1RA, glucagon-like peptide-1 receptor agonist; RYGB, Roux-en-Y gastric bypass; SG, sleeve gastrectomy; VLCD, very-low-calorie diet.

### A Risk-Stratified Framework for the Aesthetic Practitioner

We present a 6-step risk-stratified framework for nutritional awareness in GLP-1RA patients presenting for aesthetic consultation (Figure 2); the sequential clinical actions and key considerations for each step are summarized in Table 4. The framework targets at-risk patients, not universal screening, and awaits prospective validation.

**Figure 2.**
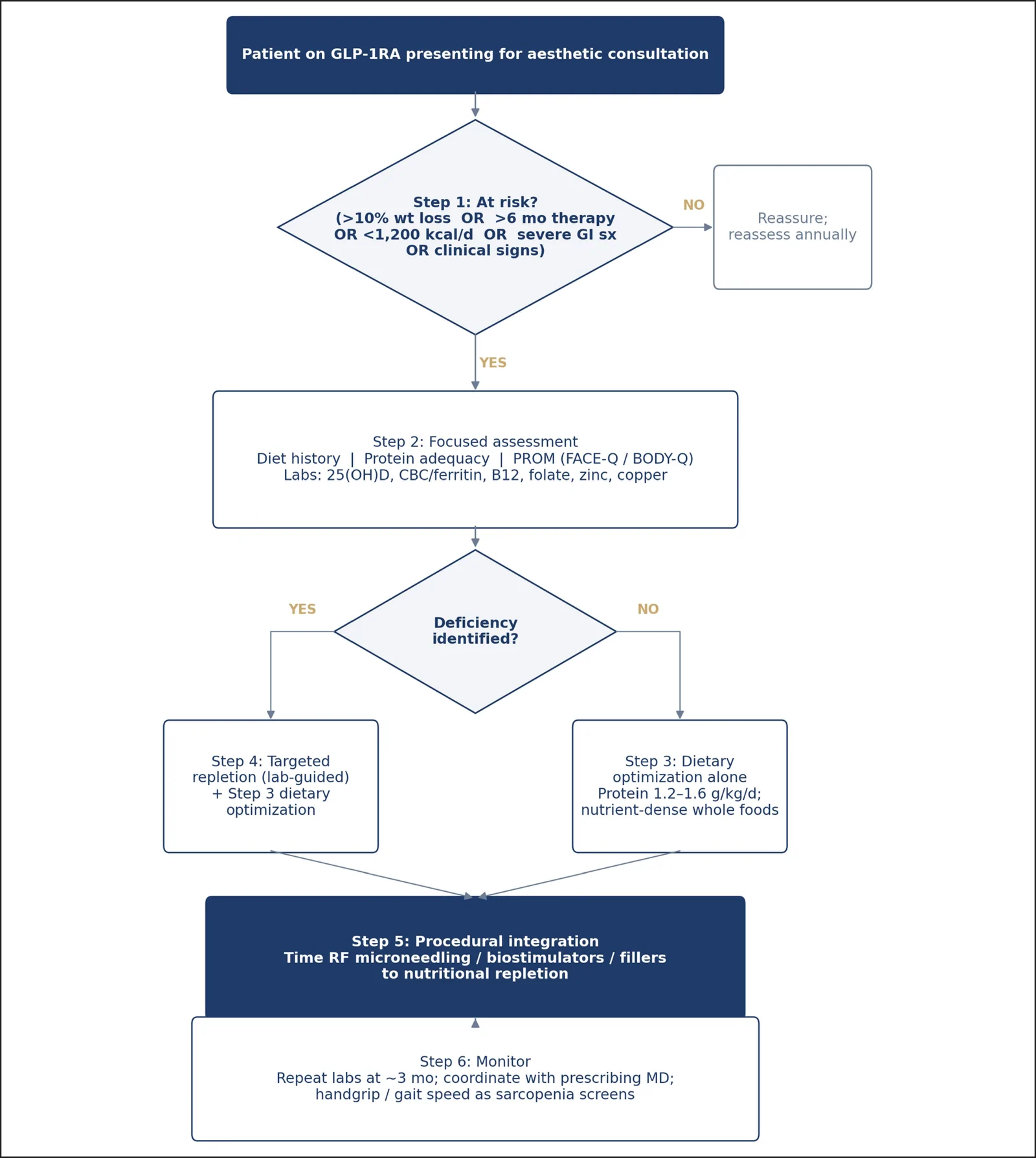
Clinical algorithm operationalizing the proposed 6-step, risk-stratified framework for patients receiving GLP-1 receptor agonist therapy who present for aesthetic consultation. Patients meeting Step 1 risk criteria proceed to focused dietary, functional, and laboratory assessment; subsequent management is determined by the presence or absence of documented deficiency. Procedural interventions are timed to nutritional repletion when deficiencies are present. BODY-Q, Body-Q; CBC, complete blood count; FACE-Q, Facial Assessment and Cosmetic Enhancement Questionnaire; GI sx, gastrointestinal symptoms; GLP-1RA, glucagon-like peptide-1 receptor agonist; MD, physician; PROM, patient-reported outcome measure; RF, radiofrequency.

**Table 4.** Risk-Stratified Framework for Nutritional Awareness in GLP-1RA Patients

Step	Clinical actions	Key considerations
1: risk stratification	>10% weight loss, >6 months therapy, <1200 kcal/day, GI symptoms, clinical signs	Low-risk patients: no routine laboratory screening
2: focused assessment	Dietary intake history (source of caloric reduction: prefer cuts from refined starch/fat, preserve protein/vegetables); protein adequacy; PROM screening (FACE-Q/BODY-Q)	Laboratories if indicated: 25(OH)D, CBC/ferritin, B12, folate, zinc, copper
3: dietary optimization	Whole-food emphasis: protein 1.2-1.6 g/kg/day; vitamin C–rich produce; zinc/iron/copper foods	RD referral if <1200 kcal/day or limited variety
4: supplementation (routine + targeted)	Routine: vitamin D 1000-2000 IU/day and omega-3 1-2 g/day EPA + DHA. Laboratory-guided: iron, B12, zinc/copper. Oral nutrition supplements ± collagen peptides if protein intake inadequate (hypothesis-generating)	Not routine; dietary optimization is foundational
5: procedural integration	Optimize nutrition before RF microneedling, biostimulators; stage filler with dietary plan	Energy-based collagen remodeling is cofactor-dependent
6: monitoring	Reassess labs at ∼3 months; coordinate with prescribing physician; dietitian for persistent issues	Handgrip/gait speed as practical sarcopenia screens

Proposed 6-step, risk-stratified framework. Framework is based on synthesis of current evidence and awaits prospective validation. CBC, complete blood count; GLP-1RA, glucagon-like peptide-1 receptor agonist; PROM, patient-reported outcome measure; RD, registered dietitian.

#### Step 1: Identify At-Risk Patients

Criteria include weight loss exceeding 10% of body weight; GLP-1RA duration >6 months; caloric intake below 1200 kcal/day; severe or persistent gastrointestinal symptoms resulting in food avoidance; or clinical signs of nutritional compromise (fatigue, hair thinning, and poor wound healing). The Ozempic-face presentation itself—temporal wasting, malar flattening, and periorbital hollowing following rapid weight loss—warrants focused nutritional assessment, as such patients by definition meet the weight-loss criterion. Low-risk patients do not require routine laboratory screening.

#### Step 2: Focused Assessment

Dietary intake history should characterize not only total caloric intake and protein consumption (target 1.2-1.6 g/kg/day) but the source of caloric reduction: ideally, the reduction is drawn from refined starches and added fats, whereas protein, vegetables, fruit, and other nutrient-dense foods are preserved or increased in proportional terms. Food variety, meal pattern, and tolerability of high-volume foods should be reviewed. Laboratory evaluation when indicated: 25-hydroxyvitamin D, complete blood count (CBC) with ferritin/iron, vitamin B12, folate, zinc, and copper. A brief patient-reported outcome instrument (eg, FACE-Q Appearance-related Psychosocial Distress or BODY-Q weight-related body-image scales) can screen for post-weight-loss appearance distress that may benefit from counseling concurrent with procedural planning.

#### Step 3: Dietary Optimization (Foundation)

The primary intervention is nutrient-dense dietary optimization. Patients should be actively educated on what a nutrient-dense, protein-forward diet looks like at a reduced caloric ceiling, because each remaining calorie must now carry greater micronutrient density. Registered dietitian referral is strongly encouraged when intake is below 1200 kcal/day or food variety is limited. Emphasize overall vegetable and fruit intake for antioxidants, citrus and leafy greens for vitamin C; lean meats, shellfish, and legumes for zinc and iron; fatty fish and dairy for vitamin D; organ meats, shellfish, and nuts for copper; and high-quality, leucine-rich protein. This is particularly important during active weight loss to mitigate lean-mass loss.

#### Step 4: Supplementation (Routine and Targeted)

Two supplements may reasonably be offered routinely to GLP-1RA patients regardless of baseline laboratory values, given high background prevalence of insufficiency and a favorable risk–benefit profile: vitamin D, 1000-2000 IU/day (titrating per Endocrine Society guidelines when 25(OH)D values are available), and omega-3 fatty acids, 1 to 2 g/day eicosapentaenoic acid (EPA) + docosahexaenoic acid (DHA), which support membrane lipid composition, anti-inflammatory eicosanoid balance, and dermal barrier integrity.^46^ The remaining supplements should be laboratory-guided rather than routine: iron only if deficiency is documented; vitamin B12 if serum is low; and zinc 15 to 30 mg with copper 1 to 2 mg if confirmed deficient. In patients whose whole-food protein intake remains inadequate despite counseling, oral nutrition supplements (15-30 g protein per serving) and oral hydrolyzed collagen peptides (5-15 g/day, co-administered with vitamin C) have shown skin elasticity benefits in non-GLP-1RA populations; no studies have evaluated this intervention in GLP-1RA patients, and this recommendation should be considered hypothesis-generating rather than evidence-based.^42,43^

#### Step 5: Procedural Integration

Energy-based collagen remodeling procedures (eg, radiofrequency microneedling) should be preceded by nutritional optimization to maximize procedural yield, as neocollagenesis is cofactor-dependent. Volume restoration may be staged alongside nutritional optimization.

#### Step 6: Monitoring and Coordination

Reassess abnormal laboratory values at ∼3 months. Communicate findings to the prescribing physician. Refer to dietitian for persistent deficiencies, intake below 1000 kcal/day, or sarcopenia signs. Handgrip dynamometry and gait speed serve as practical screening adjuncts.^24^

### Implementation Considerations

The proposed laboratory panel (25-hydroxyvitamin D, CBC with ferritin, vitamin B12, folate, zinc, and copper) costs ∼US$120 at Medicare and commercial reimbursement rates and requires a single venipuncture.^47^ Anticipated yield scales with risk tier: near-zero clinically relevant abnormalities in low-risk patients (Butsch cohort baseline rate ≈2%) vs ≈20% to 30% expected yield in high-risk patients meeting ≥2 Step 1 criteria. Broad adoption without risk stratification risks overtesting and medicalization and should therefore be discouraged.

## DISCUSSION

GLP-1RA therapy may create a nutritional–dermal mismatch in which tissue-remodeling demand increases while the cofactors required to complete that remodeling decline. The degree to which this mismatch translates into measurable skin impairment—distinct from the expected consequences of rapid weight loss of any etiology—has not been established prospectively.

The pathophysiology of GLP-1RA-related facial aesthetic change is multifactorial: (1) mechanical, through fat depletion; (2) structural, through collagen disorganization; (3) nutritional, through cofactor depletion; and (4) potentially pharmacological, through direct GLP-1 receptor effects on target cells, although this remains unconfirmed.

Several caveats temper the framework. Most GLP-1RA patients do not develop clinically significant deficiencies with standard monitoring; ∼78% in the Butsch cohort had no documented deficiency at 1 year.^32^ Facial changes may be primarily attributable to fat depletion rather than nutritional insufficiency. The link between subclinical micronutrient shortfalls and measurably worsened skin outcomes in GLP-1RA patients specifically remains undemonstrated. Dietary counseling alone may suffice for most patients, and formal screening cost-effectiveness is unknown; broad adoption without clinical judgment could lead to unnecessary testing and medicalization.

A central question this review cannot resolve is whether “Ozempic face” represents a pharmacologically distinct entity or simply the expected facial consequence of any rapid, substantial weight loss. The null hypothesis—that GLP-1RA-induced facial aging is indistinguishable from that seen with severe caloric restriction of any etiology—has not been refuted by current evidence. Preclinical data on direct GLP-1 receptor effects in dermal tissue raise the possibility of a drug-specific contribution, but these findings remain in vitro and have not been confirmed in human skin at therapeutic drug concentrations.^44^

Recommendations developed in the obesity-medicine setting have direct relevance to the aesthetic practitioner who encounters these patients downstream. Almandoz et al recommended that clinicians identify preexisting nutritional risk factors before initiating antiobesity medications and that ongoing monitoring should address protein adequacy, micronutrient intake, gastrointestinal symptoms, and fluid status.^35^ Their structured approach to pretreatment screening, dietary quality optimization, and interval monitoring provides the conceptual foundation upon which the aesthetic-specific framework presented here is built. We extend these recommendations by considering how cofactor depletion during active dermal remodeling may compound the visible consequences of rapid weight loss on the face.

For patients who experience psychological distress at appearing older after weight loss, nutritional optimization and procedural intervention can be framed as complementary strategies that align appearance with improved health.^6,9^ Brief patient-reported outcome measure (PROM) screening (Step 2) supports identification of patients in whom psychological support should accompany procedural care.

### Limitations

Several limitations should be considered when interpreting this review. Most GLP-1RA-related nutritional-deficiency data are observational or claim-based rather than derived from prospective, systematically screened cohorts; reported deficiency rates may therefore reflect differences in baseline diet, comorbidity, medication dose, gastrointestinal adverse effects, duration of therapy, and testing practices rather than a uniform drug effect, and true prevalence—particularly of subclinical insufficiency—is likely underestimated. Evidence for direct GLP-1RA effects on dermal cells remains preclinical and largely confined to in vitro studies using supraphysiologic agonist concentrations; current evidence therefore cannot reliably separate a drug-specific dermal effect from the facial consequences of rapid weight loss of any etiology.^8,44^

The proposed 6-step risk-stratified framework has not undergone prospective validation. Its thresholds are extrapolated from the obesity medicine, nutrition, and aesthetic-surgery literature rather than derived from GLP-1RA-specific aesthetic cohorts, and objective endpoints—standardized 3D facial imaging, cutometry, corneometry, longitudinal micronutrient testing, and weight-matched controls—will be needed before routine screening or supplementation can be recommended beyond individualized clinical judgment.

Finally, this narrative review carries methodologic limitations inherent to its design: non-English articles were excluded during screening because dual-reviewer translation resources were not available, which may have omitted relevant regional data, and, as with any narrative synthesis, the selection and interpretation of evidence carry inherent interpretive latitude that a systematic review or meta-analysis would more rigorously constrain.

### Future Research Directions

Six directions are prioritized. (1) A prospective cohort of GLP-1RA initiators with serial cutometry, corneometry, standardized 3D facial imaging, and micronutrient panels (vitamin C, zinc, copper, B12, 25(OH)D, and ferritin) at 0, 3, 6, and 12 months, with weight-matched nonpharmacologic comparators, to test the mismatch hypothesis against the null. (2) A double-blind randomized trial of micronutrient-targeted repletion (with or without hydrolyzed collagen) vs standard counseling in GLP-1RA patients with documented deficiency, with dermal biopsy and validated patient-reported outcome endpoints. (3) Ex vivo human skin explant studies of therapeutic-concentration semaglutide and tirzepatide on ADSC differentiation, fibroblast COL1A1/COL3A1 expression, and dWAT lipolysis, to bridge existing in vitro data to human tissue at physiologically achievable concentrations. (4) A Markov cost-effectiveness model of the proposed 6-step framework, reporting incremental cost per deficiency detected and per unit improvement in facial PROMs, to inform guideline adoption and payer coverage. (5) A randomized trial of structured resistance and whole-body exercise—with a parallel pilot arm of facial muscle exercise—initiated concurrently with GLP-1RA therapy, with co-primary endpoints of lean-mass preservation (DXA) and skin biophysical and PROM measures, to disentangle the contributions of activity-mediated lean-mass preservation from nutritional substrate availability. (6) A longitudinal stool and skin microbiome cohort under GLP-1RA therapy with correlation to skin biophysical, dermatologic, and PROM outcomes, to test whether GLP-1RA-induced shifts in gut microbial composition (and resulting changes in short-chain fatty acid and bile-acid profiles) modulate dermal repair capacity.

## CONCLUSIONS

Patients seeking GLP-1RA therapy and those presenting for aesthetic surgery are frequently the same individuals, motivated by a shared goal of aligning appearance with improved health. Aesthetic practitioners are uniquely positioned to recognize the nutritional consequences that may accompany pharmacologically induced weight loss. Although direct causal pathways from GLP-1RA therapy to specific nutritional deficits to measurable skin outcomes have not been established, this review provides a practical, risk-stratified approach for the aesthetic practitioner: whole-food dietary optimization as the foundation, routine supplementation of vitamin D and omega-3 fatty acids in selected patients, laboratory-guided repletion reserved for documented deficiencies of iron, B12, and zinc/copper, and procedural timing coordinated with nutritional status. Prospective studies are now needed to determine whether targeted nutritional strategies translate into measurable improvements in skin quality and procedural outcomes in this rapidly growing patient population.
